# Social perceptions, age stereotypes, and health behaviours: an economic and public health perspective from China

**DOI:** 10.3389/fpubh.2026.1727494

**Published:** 2026-01-30

**Authors:** Ying Zhou, Yixin Deng, Yijia Liu

**Affiliations:** 1 School of Economics and Finance, Hohai University, Changzhou, China; 2 Department of International Business and Trade, Kyung Hee University, Seoul, Republic of Korea

**Keywords:** health behaviour, health inequality, healthy ageing, social climate, social cognition, stereotypes, urban–rural disparity

## Abstract

Population ageing is accelerating worldwide, making public attitudes towards older adults an increasingly important concern for public health. Using nationwide survey data from Chinese adults across multiple provinces, this study applies a multilevel analytical framework in which individual health behaviours are examined within provincial social climates, allowing both personal perceptions and contextual conditions to be considered simultaneously. The analysis focuses on two parallel pathways, namely the association between positive social cognition of ageing and health behaviour, and the association between age-related stereotypes and behavioural engagement, while also assessing how provincial discourse on ageing conditions these relationships. Results show that more favourable perceptions of ageing are strongly associated with higher levels of preventive care, physical activity, and social participation, with standardised coefficients ranging from approximately 0.46 to 0.55. About one quarter of this association is linked to an indirect pathway, whereby stronger social cognition corresponds to lower endorsement of negative stereotypes, which in turn is associated with healthier behaviour. Age-related stereotypes display a consistent negative association with health behaviour, with coefficients between −0.36 and −0.41, and this pattern is particularly pronounced in rural areas. Contextual conditions further shape these associations. In provinces characterised by more supportive public discourse on ageing, the link between cognition and behaviour is stronger, whereas in environments where stereotypes are more salient, their negative association with behaviour is amplified. Urban residents show a stronger alignment between positive views of ageing and behavioural engagement than rural residents, indicating an institutional gradient in healthy ageing. Taken together, the findings suggest that health behaviour in later life reflects not only individual orientations but also the social narratives and institutional environments surrounding ageing, implying that policies aimed at promoting healthy ageing may benefit from combining individual-level education with broader efforts to improve public discourse on ageing and to reduce persistent urban–rural inequalities in health systems.

## 1 Introduction

We apologize for the oversight. Due to an incorrect file upload, the previously submitted version did not display the in-text citations in the Introduction; the entire Introduction has therefore been replaced with an identical version in which references (27–41) are now properly cited, without any substantive changes to the text:Since the beginning of the twenty-first century, global population ageing has accelerated markedly. According to the United Nations, adults aged 65 and over accounted for more than 10 percent of the world’s population in 2024 and are projected to reach 16 percent by 2050 (27). China’s demographic transition has proceeded even more rapidly and with greater complexity than the global average. By the end of 2023, China had 297 million people aged 60 and above, accounting for 21.1 percent of the total population, including more than 210 million aged 65 and above (28). This unprecedented scale of ageing presents enduring challenges for economic growth, social protection, and public health, and has prompted renewed attention to how ageing is understood and managed in society.Population ageing itself is not the core problem; rather, its consequences depend on how older people are perceived and treated. In this respect, social cognition of ageing and age-related stereotypes are central. Social cognition refers to how individuals and communities perceive population ageing, interpret ageing-related policies, and evaluate the social roles, responsibilities, and contributions of older adults, while age-related stereotypes consist of widely shared assumptions about older people’s competence, adaptability, productivity, and dependence (41). These perceptions are embedded in everyday interaction and institutional practice, rather than existing as abstract attitudes. Stereotypical labels, such as viewing older adults as conservative, disconnected, or inevitably frail, gradually erode both the public roles older citizens are able to occupy and their confidence in shaping their own futures. Through repeated social encounters, such shared perceptions can be internalised, influencing choices related to diet, physical activity, medical care, and social engagement (39).Academic perspectives on ageing have evolved considerably over time. Earlier research often equated later life with physical and cognitive decline, with work in the 1980s focusing on memory loss and slower cognitive processing (29, 30). Subsequent studies reframed ageing as a process involving adaptive neural and cognitive change, highlighting the role of neuromodulation and representational reorganisation (31, 32). Later concepts such as healthy cognitive ageing and dementia prevention further emphasised the importance of maintaining cognitive resources across the life course (33).More recent scholarship situates ageing within networks of social relationships and cultural contexts, examining how social visibility, isolation, and shared perceptions shape cognition, well-being, and behaviour in later life (34). In China, research has increasingly moved beyond the individual level to consider how broader social environments influence ageing and health. Living near parks, breathing cleaner air, and residing in favourable climatic conditions have been linked to stronger cognitive performance in mid and later life (36). At the community level, denser social networks and higher levels of participation are associated with better cognitive outcomes among older adults, reflecting the role of local social capital in shaping ageing experiences (37). By contrast, residential segregation and limited local infrastructure can exclude older adults from social life and health-promoting routines. Evidence from the United States shows that such forms of spatial and social exclusion are associated with poorer cognitive and emotional outcomes in later life, a pattern that is highly relevant to China’s rapidly urbanising context (38).At the institutional level, broader coverage and accessibility of social health insurance can buffer the adverse health consequences of population ageing by reducing barriers to care and supporting preventive behaviour among older adults (40). Taken together, these findings suggest that health disparities in later life cannot be attributed solely to biological ageing, but are also shaped by societal perceptions of ageing, economic resources, and the availability of local health services. Despite this progress, important gaps remain. Most existing studies focus on single-level determinants and lack an integrated framework linking individual cognition, community context, and institutional conditions. Age-related stereotypes are often treated as outcomes rather than as mechanisms connecting social environments to behaviour, and public-health and economic factors are frequently discussed descriptively rather than tested within rigorous multilevel models (35). To address these limitations, this study examines how social cognition of ageing and age-related stereotypes are associated with individual health behaviours in China, and how these relationships vary across different social environments. Using nationwide survey data and a multilevel framework that nests individuals within provincial contexts, the study integrates perspectives from economics and public health to explore how shared perceptions of ageing, local social conditions, and institutional settings jointly shape patterns of health behaviour.

## 2 Review

### 2.1 Current research on ageing, social perceptions, and health behaviour

The accelerating pace of population ageing has encouraged scholars to move beyond an exclusive focus on biological decline and to pay increasing attention to the social and behavioural dimensions of ageing. Miyawaki et al. (1), in a scoping review, observed that the concepts of “cognitive ageing” and “cognitive health” remain inconsistently defined across disciplines, noting that differences in measurement traditions and conceptual frameworks have made it difficult to compare findings and integrate results across studies. Piccinin et al. (2) further emphasised that meaningful understanding of cognitive change requires longitudinal evidence and analytical approaches capable of tracing how cognitive function evolves over time, rather than relying solely on cross-sectional contrasts. Within the Chinese context, a growing body of research has examined how social and residential environments shape cognitive outcomes. Shi et al. (3) found that age-friendly urban design and community development are closely associated with better cognitive performance among older adults and that accessible, well-designed public facilities may slow cognitive decline. Extending this perspective, Ko (4) highlighted the importance of neighbourhood environments and household conditions, suggesting that feelings of safety and belonging may serve as important mediating pathways. Using national survey data, Luo et al. (5) similarly reported that community attachment and residential quality are directly related to the pace of cognitive deterioration, while Liu et al. (6), applying multilevel modelling, showed that community-level socioeconomic status is strongly associated with individual cognitive functioning. These findings indicate that cognitive ageing is shaped not only by individual characteristics but also by broader social and environmental contexts. Evidence from cross-national comparisons provides further support for this view. Ailshire and Zhang (7) demonstrated that differences in social institutions and cultural norms substantially influence older adults’ health and well-being, underscoring the importance of situating China’s demographic ageing within an international perspective. Social engagement and interpersonal connection have likewise been recognised as central to maintaining cognitive health. Longitudinal research by Zhou et al. (8) found that sustained participation in social activities is associated with a significantly lower risk of cognitive impairment in later life, a pattern echoed by Fu et al. (9), who showed that frequent social participation is linked to better cognitive performance. More recent work by He et al. (10) noted that digital transformation has reshaped patterns of social engagement, with traditional media use, online connectivity, and social-media interaction emerging as new arenas of participation. These evolving forms of engagement appear to strengthen social ties among older adults and, in turn, help sustain cognitive functioning. Taken together, this body of research outlines a multidimensional framework in which ageing, social cognition, and health behaviour are closely intertwined, providing both theoretical and empirical foundations for examining age-related stereotypes and health behaviour patterns in China’s rapidly ageing society.

### 2.2 The contributions and limits of social and environmental factors to cognition and health behaviour

Social and environmental conditions are widely recognised as shaping both cognitive health and health-related behaviour in later life. Early work by Fisher et al. (11) proposed a multilevel framework linking social and physical environments to individual activity patterns and behavioural outcomes. Although this model has remained influential, it has seldom been applied to empirical research on cognitive ageing, and direct evidence supporting its mechanisms has remained limited. More recent studies have begun to address this gap. Rodrigues and Delerue-Matos (12), in their review of research on social exclusion and cognition, documented a consistent association between social isolation and cognitive decline. Their synthesis contributed important insight into the social and psychological dimensions of ageing, but relied primarily on cross-sectional evidence, which constrained interpretation of temporal processes. Environmental influences have received comparable attention. Li et al. (13) showed that exposure to air pollution, particularly elevated ozone concentrations, is associated with a higher risk of Alzheimer’s disease and mild cognitive impairment, as well as increased economic burden. Because their analysis was based largely on environmental monitoring and burden-of-disease estimates, however, it provided limited information on how cognitive change unfolds within individuals. Some of these limitations have been partly addressed by longitudinal research. Using a national cohort, Zu et al. (14) identified a complex configuration of influences on cognitive function, including environmental hazards, socioeconomic position, and everyday lifestyle factors. Their findings offered a more comprehensive empirical account of cognitive ageing. Socioeconomic status has also been repeatedly identified as a key dimension of healthy ageing. Meixia et al. (15) reported that lower levels of education, income, and social resources are associated with a higher risk of cognitive impairment, although their study did not explore whether social support mediates these relationships. Related evidence comes from Yang et al. (16), who examined populations relocated from poverty-stricken areas and found that strong community networks were associated with lower depressive symptoms and indirectly supported the maintenance of cognitive functioning. Together, these findings suggest that social connections can play a compensatory role under conditions of economic disadvantage. Physical functioning and everyday health behaviour form another important link between social context and cognition. Qi et al. (17) demonstrated that regular physical activity is associated not only with improved physical fitness but also with better cognitive performance, pointing to a mutually reinforcing relationship between behaviour and cognitive health. Their analysis, however, paid limited attention to how opportunities for physical activity differ across social environments, leaving open the question of how contextual inequalities shape these behavioural pathways. Existing research provides substantial evidence that social and environmental conditions influence cognitive and behavioural ageing through multiple channels. At the same time, much of this literature remains cross-sectional or conceptually driven, and the mechanisms linking social context, behaviour, and cognition are not yet fully clarified. Recent studies have increasingly moved beyond single-factor explanations towards multilevel perspectives that integrate individual, community, and structural influences. This shift allows for a more realistic understanding of ageing processes and establishes a stronger empirical basis for research that can inform policy development and intervention design.

### 2.3 The mechanisms linking social engagement, social capital and healthy ageing

In studies of healthy ageing, social participation and social capital have emerged as key factors that sustain cognitive function and psychological well-being. Jiang et al. (18), drawing on cross-national evidence, found that older adults living in communities with stronger social capital show better cognitive performance. The effect was especially notable in low- and middle-income countries, suggesting that social connections can partly offset material disadvantage when financial or institutional resources are scarce. Perkins and Ball (19) proposed a relational framework that situates ageing, illness, and care within the network of social relationships. Their work highlights that interpersonal bonds do more than provide emotional comfort; they help older adults manage health decline and the demands of long-term or end-of-life care, thereby delaying negative outcomes. Research in China has reached similar conclusions. Ye and Zhang (20) reported that different social network structures produce distinct health profiles among rural older adults, with social support acting as a key mediating pathway. Their findings suggest that social capital should not be viewed as a fixed set of resources but rather as a process that evolves through ongoing exchange, continuously shaping cognitive and physical health. Jing et al. (21) added that social support mediates the relationship between cognitive function and depression, and that the strength of this mediation varies across age groups among those already experiencing depressive symptoms. Taken together, these studies show that social participation and social capital influence cognition and health through multiple, intertwined mechanisms: the practical help of social support, the emotional closeness embedded in relationships, and the reciprocal exchanges that unfold within social networks. This body of evidence illustrates how ageing can remain cognitively and mentally healthy when grounded in strong social connection and community engagement.

### 2.4 Evolution of research methods and theoretical frameworks: advances and remaining gaps

Research on ageing, cognition, and health behaviour has made steady progress in both methodological sophistication and theoretical scope, yet important limitations persist. Roheger et al. (22), in a systematic review and meta-analysis drawing on longitudinal evidence and standardised quantitative instruments, demonstrate that the transition from healthy ageing to Alzheimer’s disease is accompanied by a discernible trajectory of social cognitive impairment. Their synthesis offers valuable insight into the neurodegenerative continuum, but it focuses almost entirely on biological and cognitive decline, leaving the role of social context and everyday health behaviour in shaping this trajectory largely unexplored. As a result, the review provides only a partial picture of how societal conditions intersect with biological change over time. A complementary perspective is offered by McAnally and Hagger (23), whose meta-analysis confirms a strong association between health literacy and behavioural outcomes, supplying robust evidence that individual-level cognitive processing is closely linked to health practices. At the same time, their analysis centres primarily on cognitive constructs and decision-making at the individual level, paying limited attention to broader social structures, environmental inequalities, or institutional constraints that may condition these relationships. Tomás and Ravazzini (24) approach the issue from a different angle by proposing an inclusive mixed-methods design that combines quantitative estimates with qualitative accounts of older adults’ everyday practices across multiple countries. This integration is intended to compensate for the limitations of single-method approaches, yet the study is constrained by relatively small samples and substantial cross-cultural variation, which complicates the transferability of its findings to the large and heterogeneous context of China. Using Chinese data, Li and Xu (25) adopt a longitudinal design to show that shifts in patterns of social participation are associated with improvements in cognitive functioning among middle-aged and older adults, underscoring the value of repeated measurement for capturing dynamic relationships. Nevertheless, their reliance on structured survey instruments limits insight into the quality of social ties and the texture of daily interaction, leaving the underlying social mechanisms only partially specified. Taken together, these studies reflect significant advances in ageing research, but they also reveal a common tendency to operate within a single analytical level. As a result, integrative accounts that trace cross-level mechanisms linking social conditions, cognitive processes, and health behaviours remain rare, hindering the development of a coherent framework capable of explaining how these domains mutually shape one another over time.

### 2.5 Research gaps and the innovative positioning of this study

Although research on ageing, social cognition, and health behaviour has expanded rapidly, important gaps remain. Rodrigues and Delerue-Matos (12), in a systematic review, found that social exclusion heightens the risk of cognitive decline among middle-aged and older adults. Yet most of the studies they examined were cross-sectional, leaving the direction of causality uncertain. The longitudinal work of Zu et al. (14) helps to address this weakness by showing that cognitive function reflects a network of interrelated influences. Even so, their analysis stops short of explaining how social context, economic constraint, and health behaviour interact and shape one another. Within the narrower field of health literacy, McAnally and Hagger (23) reported through meta-analysis a strong association between literacy and health behaviour. Their review, however, focuses on individual cognition and does not extend to cultural or structural settings. At a broader scale, Ailshire and Zhang (7) demonstrated that national institutions and cultural climates leave distinct marks on late-life health, suggesting that single-level models fail to capture the complexity of person–environment relations. Similar limitations appear where public-health and economic analyses meet. Li et al. (13) estimated the economic burden of cognitive impairment caused by environmental pollution but did not test whether social capital or health behaviour mediates that link. Meixia et al. (15) showed that socioeconomic position explains a large share of health inequality in later life, yet they did not embed economic disadvantage within the wider network of psychosocial factors. Earlier still, Levy (26) reminded us that social cognition does not merely reflect external reality: self-stereotypes can influence personal health practices. Systematic evidence on how stereotype activation, cognition, and behaviour interact remains limited, particularly in China, where population ageing is accelerating.

To respond to these gaps, the present study pursues three aims.

First, it brings social cognition and stereotype processes into the analysis of health behaviour and examines their dynamics through both economic and public-health perspectives.

Second, situated within China’s demographic transition, it provides context-specific evidence that contributes to international comparative work.

Third, it builds an interdisciplinary framework to explore how social environments, economic structures, and individual cognition interact. This integration seeks to move beyond single-level explanations and to give policy and practice a firmer scientific base for promoting healthy ageing.

Over the past two decades, research on ageing, social cognition, and health behaviour has gradually evolved into a multidimensional field. Early studies concentrated on measuring cognitive ageing and improving methodology; later work turned to the roles of social participation, neighbourhood conditions, and social capital; and more recent contributions have expanded to cross-national comparison and environmental risk. These developments have produced a rich foundation of theory and data. Methods have advanced as well—longitudinal designs, meta-analyses, and mixed approaches have all added depth—yet integration across analytical levels is still incomplete. Building on this accumulated knowledge, three issues remain especially salient. The causal sequence linking social context and health behaviour is still unclear. Economic and public-health dimensions are often examined separately rather than jointly. And the connection between age stereotypes and cognitive health has received too little systematic testing. These gaps define the starting point for the present study and highlight the need, in China’s rapidly ageing society, for a more comprehensive approach that joins social cognition, health behaviour, and the intertwined realities of economy and public health.

## 3 Empirical strategy

### 3.1 Research design and participants

**Figure 1:**
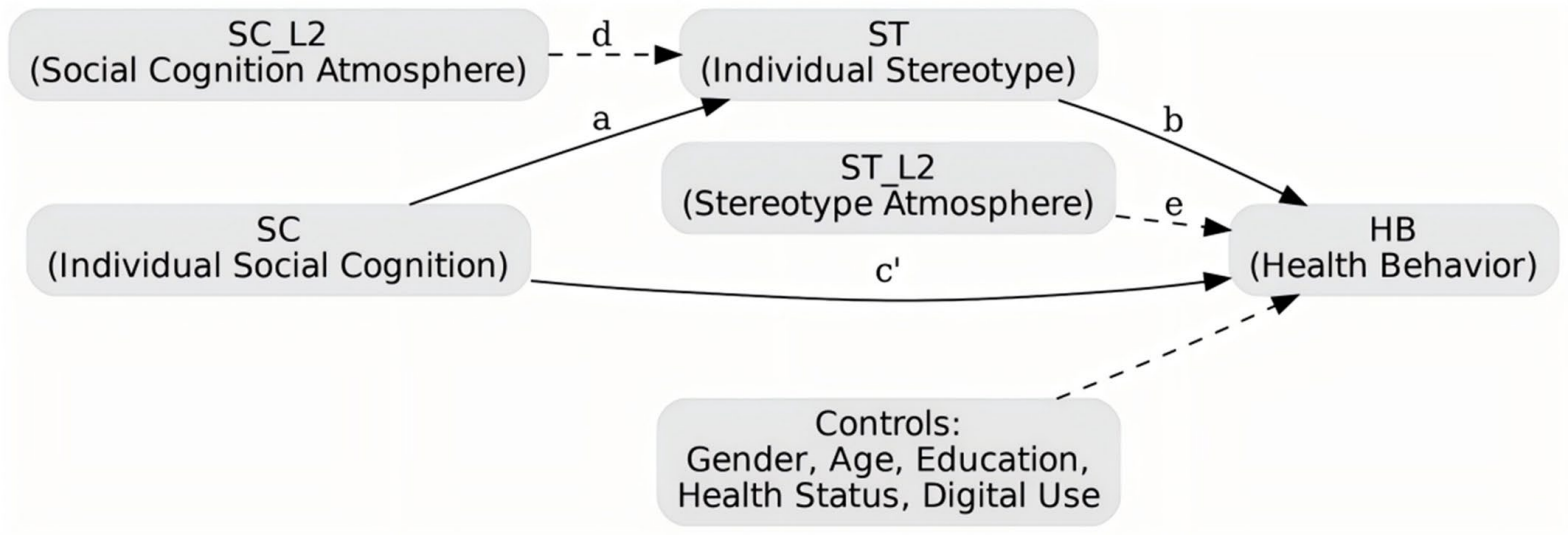
Research framework diagram.

This study uses a cross-sectional survey to explore how ageing-related perceptions and stereotypes in Chinese society influence individual health behaviours. It also examines the multilevel pathways that link these cognitions to behavioural outcomes from both economic and public-health perspectives. A multilevel linear model was adopted, with individuals (Level 1) nested within provinces (Level 2). This structure allows estimation of provincial differences in the climate of ageing cognition and stereotyping, as well as their cross-level effects on personal health practices. The study employed a stratified cluster sampling strategy covering eastern, central, western, and north-eastern regions, ensuring that the dataset reflects China’s socio-economic and regional diversity. Data were collected through a combination of online and offline surveys. For the offline part, the research team worked with physical education departments at several universities and with local community organisations. Printed questionnaires were distributed during breaks in training sessions or community events and completed on site under the supervision of trained assistants who collected them immediately. The online survey was distributed through official channels such as workplace chat groups, university portals, and community notice boards, coordinated by the same partner institutions. To maintain data quality, each participant could submit only once, and all questions were mandatory. Of the 540 questionnaires distributed, 500 were returned. Forty were excluded because of implausibly short completion times (under 120 s), logical inconsistencies, duplication, or missing information on key items. The remaining 500 valid responses yielded an effective response rate of 92.6%. The sample is balanced across China’s macro-regions, ensuring sufficient statistical power at the provincial level. All respondents were residents of mainland China aged 18 or above and reported their province, type of locality (city, town, or rural village), and setting (community, workplace, school, or care facility). Province was treated as a random intercept at Level 2, while locality type and setting were included as control variables to account for contextual differences in health behaviour. Figure 1 illustrates the nested, multilevel design of the study. Individual-level variables—social cognition, stereotypes, health behaviour, and controls—are positioned at Level 1, and the corresponding provincial climates of cognition and stereotyping are located at Level 2. The arrows represent direct, cross-level, and mediating effects. Together, these elements form an empirical framework that combines economic and public-health perspectives in analysing the determinants of healthy ageing. Although the sample includes respondents from multiple provinces and all major macro-regions of China, it is not a fully probability-based national sample. The results should therefore be interpreted as analytically representative rather than strictly representative of the national population, and caution is needed when generalising population-level prevalence.

### 3.2 Measurement instruments

The questionnaire is organised into five sections that collect control variables, predictors, mediators, outcomes and contextual moderators.

(1) Basic information and controls cover sex, age, education, marital status, employment, chronic conditions, household structure and frequency of digital technology use, allowing individual heterogeneity to be held constant.
(2) Social cognition (SC) is measured with a five-point Likert scale that taps respondents’ views on population ageing, related policies and the changing social role of older people in China. Two “group-referent” items are included so that individual scores can be aggregated to create a level-2 measure of perceived social-cognitive climate (SC_L2).

Social cognition concerns how individuals perceive and interpret ageing-related issues in society, rather than objectively testable factual knowledge. For this reason, self-reported measures are appropriate for capturing these subjective evaluations. To reduce the influence of social-expectation pressures, the survey was administered anonymously and did not collect any identifying information. Item wording was kept descriptive and neutral, avoiding language that might signal socially desirable positions. In addition, the inclusion of group-referent items directs respondents to evaluate the broader social environment rather than to state personal approval, which helps lower the tendency towards self-presentation. Although social-desirability bias cannot be fully excluded, these design choices are more likely to dampen extreme responses than to exaggerate observed relationships.

(3) Stereotypes towards older people (ST) are assessed with Likert items that describe both positive and negative aspects of older adults’ competence, contribution and social utility. Two group-referent items are again provided, permitting the construction of a level-2 stereotype climate variable (ST_L2).

Because attitudes towards ageing can be socially sensitive, responses may be shaped by a desire to appear tolerant or respectful. To limit this tendency, the stereotype items were constructed to cover a broad range of everyday characterisations rather than overtly evaluative judgements. Several items refer to commonly observed social views rather than personal beliefs, allowing respondents to answer with less self-consciousness. The inclusion of both positive and negative descriptions further encourages differentiated responses instead of uniform endorsement of socially approved views.

(4) Health behaviour (HB) is captured across five domains: physical activity, dietary habits, sleep routine, preventive check-ups and social participation. All items use a common time anchor (“past 7 days” or “past 30 days”) and a graded frequency response format.

Health behaviour is operationalised using frequency-based measures rather than indicators of intensity or quality. This choice reflects the study’s emphasis on patterns of participation in everyday health-related activities, rather than on clinical assessments of behavioural dosage. In a nationally diverse sample that spans wide differences in age, education, and health literacy, frequency measures are easier for respondents to recall accurately and apply consistently. Collecting detailed information on intensity, such as distinguishing vigorous from light exercise, would increase cognitive burden and may introduce additional reporting error. The present strategy therefore prioritises comparability and reliability across regions.

(5) Economic and public-health variables include perceived medical financial burden (0–10 visual scale), accessibility and fairness of health services (five-point Likert scales), type of health insurance and sources of income (categorical). A single attention-check item at the end of the questionnaire is used to identify and exclude inattentive respondents, thereby safeguarding data quality.

### 3.3 Data structure and variable treatment

The data are structured in two levels. At Level 1, individuals constitute the basic units and provide the focal variables—social cognition (SC_i), social stereotyping (ST_i), and health behaviour (HB_i)—along with control factors such as sex, age, education, chronic illness, and internet use. At Level 2, provinces form the contextual units of analysis. For each province, responses to the group-referent items were aggregated to produce two contextual indicators: the provincial climate of social cognition (SC_L2) and that of stereotyping (ST_L2). These aggregated measures were used as province-level predictors in the multilevel model. During data preparation, the individual-level variables SC and ST were group-mean centred to distinguish within-province from between-province effects and to improve model interpretability. Province was included as a random intercept to account for differences in health behaviour across provinces.

### 3.4 Analytical strategy

The analysis was conducted in several stages to maintain statistical rigour. Descriptive statistics and Pearson correlations were first used to explore the relationships among the key variables. Reliability was examined with Cronbach’s alpha and McDonald’s omega, and the measurement model was tested using confirmatory factor analysis. To assess measurement equivalence across groups, configural, metric, and scalar invariance were evaluated sequentially. Next, we calculated the null-model ICC(1), ICC(2), and r_wg values to determine whether the data structure justified aggregation to the provincial level and warranted the use of multilevel modelling. During the modelling stage, four models were estimated in sequence: a Level-1 main-effects model, a Level-2 main-effects model, a mediation model, and a cross-level interaction model. Mediation was tested through bias-corrected non-parametric bootstrapping (5,000 resamples), which provided confidence intervals and significance estimates for the indirect effects. For the cross-level interactions, random-slope models were combined with the Johnson–Neyman technique to identify the range of the moderator for which the interaction reached statistical significance. The results were also visualised to aid interpretation. All analyses were conducted using SPSS 26.0 for descriptive statistics and reliability testing, Mplus 8.3 for confirmatory factor analysis and multilevel mediation models, and R version 4.2.2 for robustness checks and graphical presentation of interaction effects.

### 3.5 Indicator system

Figure 1 illustrates the multilevel structure and variable configuration used in this study. It depicts the nesting of individual-level (Level 1) and provincial-level (Level 2) variables and shows the hypothesised pathways linking social cognition, stereotypes, and health behaviour, including both mediation and cross-level moderation effects. Table 1 provides the operational definitions and measurement dimensions of all study variables, covering the key independent and dependent variables, mediators or moderators, and control covariates.

**Table 1:** Variable operationalization and measurement framework.

Level-1 indicators	Level-2 indicators
Social Cognition (X)—Core Independent Variable	Perceptions of population ageing; assessments of older adults’ social value; perceived risks associated with ageing; and attitudes towards policy support
Social Stereotypes (X)—Core Independent Variable	Competence stereotype; role stereotype; economic-value stereotype; active-ageing outlook
Health Behaviours (Y)—Outcome Variable	Physical activity routines; dietary and nutritional practices; sleep and circadian regularity; preventive medical behaviour; social participation
Individual-level economic and public-health variables (Y/C)—mediators and moderators	Income level and economic strain; health insurance coverage and reimbursement; service accessibility; perceived health equity
Control variables (C)	Demographic characteristics (sex, age, education, etc.); family structure; health status; digital engagement

## 4 Econometric specification

We tested mediation with bias-corrected non-parametric bootstrapping (5,000 resamples) to obtain the significance level and confidence interval of the indirect effect. Cross-level interaction was examined by fitting random-slope models; the Johnson-Neyman technique was then applied to locate the range of the moderator where the interaction becomes significant, and the resulting regions were plotted for visual inspection.

### 4.1 Multilevel linear model specification

(See Table A-1) Taking the two-level structure in which individual i is nested within province j, the empty model for health behaviour (HBij) is specified as follows.


$$\begin{array}{rr} {\mathrm{HB}}_{\mathrm{ij}} & =\beta_{0j}+r_{\mathrm{ij}},\;\;r_{\mathrm{ij}}∼N(0,\sigma^{2}),\\ \beta_{0j} & =\gamma_{00}+u_{0j},\;\;u_{0j}∼N(0,\tau_{00}), \end{array}$$

Within-group residual variance is denoted by σ^2^, and between-group variance of intercepts by τ₀₀. The ICC(1), ICC(2), and χ^2^ test statistics derived from this decomposition determine whether the data satisfy the prerequisites for multilevel modelling.

After the predictors are entered, the full two-level random-intercept random-slope model reads.

Level-1 individual-level model


$${\mathrm{HB}}_{\mathrm{ij}}=\beta_{0j}+\beta_{1j}{\tilde{\mathrm{SC}}}_{\mathrm{ij}}+\beta_{2j}{\tilde{\mathrm{ST}}}_{\mathrm{ij}}+\beta_{3}^{T}C_{\mathrm{ij}}+r_{\mathrm{ij}},$$

Level-2 provincial-level model


$$\begin{array}{rr} \beta_{0j} & =\gamma_{00}+\gamma_{01}{\mathrm{SC}}^{\left(L2\right)}j+\gamma_{02}{\mathrm{ST}}^{\left(L2\right)}j+\gamma_{0}Z^{⊤}\mathrm{Zj}+u_{0j},\\ \beta_{1j} & =\gamma_{10}+\gamma_{11}{\mathrm{SC}}^{\left(L2\right)}j+u_{1j},\\ \beta_{2j} & =\gamma_{20}+\gamma_{21}{\mathrm{ST}}^{\left(L2\right)}j+u_{2j}. \end{array}$$

Robustness checks augment the baseline specification by adding urban–rural status (Urban) and group type (Group) as additional controls.

Heterogeneity analyses split the sample into urban and rural sub-samples and re-estimate the model for each, allowing the magnitude of the estimated effects to be compared across the two populations.

### 4.2 Mediation model specification

To test the mediating pathway from social cognition to health behaviour via stereotype activation, we specify a 1–1–1 multilevel mediation model in which the same construct is measured at level 1 (within persons), level 1 (between persons), and level 1 (between contexts).


$$\begin{array}{rr} {\tilde{\mathrm{ST}}}_{\mathrm{ij}} & =\alpha_{0j}+\alpha_{1j}{\tilde{\mathrm{SC}}}_{\mathrm{ij}}+{\alpha_{2}}^{⊤}C_{\mathrm{ij}}+e_{\mathrm{ij}},\\ {\mathrm{HB}}_{\mathrm{ij}} & =\beta_{0j}+\beta_{1j}{\tilde{\mathrm{ST}}}_{\mathrm{ij}}+\beta_{2j}{\tilde{\mathrm{SC}}}_{\mathrm{ij}}+{\beta_{3}}^{⊤}C_{\mathrm{ij}}+r_{\mathrm{ij}}. \end{array}$$

The indirect effect:


$$\text{Indirect}=\alpha_{1.}\times \beta_{1.}$$

Province-level cluster bootstrap resampling was performed 5,000 times to estimate 95 per cent confidence intervals and to decompose the total effect into its direct and indirect components.

### 4.3 Cross-level interaction model specification

To test the cross-level moderating effect of “ambient climate × individual cognition/stereotype,” an interaction term was specified that multiplies the group-level atmospheric variable by the individual-level cognitive or stereotypic measure.


$$\beta_{1j}=\gamma_{10}+\gamma_{11}{\mathrm{SC}}^{\left(L2\right)}j+u1_{j},\;\;\beta_{2j}=\gamma_{20}+\gamma_{21}{\mathrm{ST}}^{\left(L2\right)}j+u2_{j}.$$

As follow:


$${\mathrm{HB}}_{\mathrm{ij}}=\gamma_{00}+\gamma_{10}\tilde{\mathrm{SC}}i_{j}+\gamma_{20}{\tilde{\mathrm{ST}}}_{\mathrm{ij}}\gamma_{11}({\mathrm{SC}}^{\left(L2\right)}j\times \tilde{\mathrm{SC}}i_{j})\gamma_{21}({\mathrm{ST}}^{\left(L2\right)}j\times \tilde{\mathrm{ST}}i_{j})$$

In this model:

$\gamma_{11}$ captures the extent to which the ambient social–cognitive climate moderates the path that runs from individual social cognition to health behaviour.

$\gamma_{21}$ does the same for the climate of stereotype endorsement in the link between individual stereotypes and behaviour.

Simple-slopes analysis together with the Johnson–Neyman technique was then used to locate the regions of the moderators in which the interaction attains significance, thereby mapping how individual effects shift across differing climatic levels.

## 5 Result

### 5.1 Descriptive statistics and correlation analysis

Tables 2, 3 describe the sample profile and the simple correlations among key variables. Some imbalances remain in a few distributions and several measures show limited discrimination. The sex ratio is nearly equal (men 50.4%, women 49.6%). Respondents’ ages range from 18 to over 60, and both residence type and education vary widely. However, whether these characteristics are evenly represented across regions remains uncertain. To avoid bias, later models include explicit controls for these structural differences. The mean score for individual social cognition (M = 3.55, SD = 0.26) is relatively high, suggesting broad awareness of ageing and related policy issues. In contrast, the mean for social stereotyping (M = 1.59, SD = 0.23) is lower, which may reflect social-desirability effects in self-reports or differences in how scale items align with experience across age groups. The mean score for health behaviour is moderate (M = 3.68, SD = 0.24), and the small standard deviation suggests limited variation between individuals. Correlation results show that social cognition is positively related to health behaviour (*r* = 0.59, *p* < 0.001), while stereotype threat is negatively related (*r* = −0.52, *p* < 0.001). The two predictors are also negatively correlated (*r* = −0.50, *p* < 0.001), indicating that people with stronger cognitive understanding of ageing are less likely to show stereotype activation. This pattern suggests that cognition promotes and stereotypes inhibit health behaviour. Further modelling—using structural equation and multilevel approaches—is needed to test the stability of these relationships and their potential for practical intervention design.

**Table 2:** Sample characteristics (*N* = 500).

Characteristic	Category	Frequency	Percentage (%)
Gender	Male	252	50.4
Female	248	49.6	
Age	18–30	105	21
	31–45	136	27.2
	46–60	144	28.8
	61+	115	23
Residence	Urban	180	36
	Town	160	32
	Rural	160	32
Education	Junior high or below	108	21.6
	High school	134	26.8
	College	129	25.8
	Bachelor or above	129	25.8

**Table 3:** Descriptive statistics and correlation matrix (*N* = 500).

Variable	Mean	SD	1	2	3	4
Social Cognition (SC)	3.55	0.26	1			
Stereotypes (ST)	1.59	0.23	−0.50***	1		
Health Behaviour (HB)	5	0.28	0.59***	−0.52***	1	
Public Health Perception (PH)	3.85	0.23	0.42***	−0.42***	0.28***	1

The symbol “*” indicates statistical significance at the 5% level (*p* < .05).

### 5.2 Psychometric properties

Table 4 reports the reliability and validity of the core measures. The three scales show good internal consistency: Cronbach’s *α* values are 0.84 for social cognition, 0.87 for stereotyping, and 0.85 for health behaviour. McDonald’s *ω* values are all above 0.85, suggesting strong item agreement. High reliability, however, does not always imply sound measurement. In some scales, limited coverage of the conceptual domain or overlap between items may create the appearance of “pseudo-consistency.” To address this, a confirmatory factor analysis (CFA) was conducted. The model fit was acceptable (CFI = 0.94, TLI = 0.92, RMSEA = 0.043, SRMR = 0.038), supporting the expected factor structure. Although these indices meet conventional standards, they were estimated from the combined sample. Invariance across key subgroups, such as gender and urban–rural residence, was then examined. Results indicate that configural, metric, and scalar invariance hold, allowing cautious group comparisons. Nonetheless, measurement equivalence should be interpreted carefully in view of China’s regional and socioeconomic diversity. The factorial structure appears stable, yet its generalisability may depend on local contexts. Future studies should test multiple-group or latent-class models to confirm that the instruments perform consistently across different populations.

**Table 4:** Measurement results.

Index	SC scale	ST scale	HB scale
Cronbach’s α	0.84	0.87	0.85
McDonald’s ω	0.85	0.88	0.86
CFA fit indices	CFI = 0.94, TLI = 0.92, RMSEA = 0.043, SRMR = 0.038		
Measurement invariance	Configural: acceptable; Metric: acceptable; Scalar: acceptable		

### 5.3 Group-level aggregation and multilevel assumptions

Table 5 presents the preliminary tests used to justify aggregating individual responses to the provincial level before estimating the hierarchical models. The between-province variance (*τ*₀₀ = 0.22) is smaller than the within-province variance (σ^2^ = 0.63), but it is large enough to suggest real differences among provinces. The intraclass correlation ICC(1) = 0.26 shows that roughly one quarter of the total variance in individual scores lies at the provincial level—above the conventional cutoff of 0.12 and therefore suitable for multilevel modelling. The ICC(2) value of 0.74 indicates high inter-rater reliability within provinces. The median r_wg of 0.81 further shows strong agreement among respondents in each province about the social climate, supporting the use of this measure as a contextual variable. The design effect of 3.6, which exceeds the threshold of 2, confirms the presence of within-cluster similarity. Ignoring this structure and fitting a standard OLS model would underestimate standard errors and inflate Type-I error rates. Overall, these statistics support the decision to aggregate province-level indicators of social-cognitive and stereotype climates and to use a multilevel specification for subsequent analysis.

**Table 5:** Aggregation and multilevel model prerequisites.

Index	Value
Between-group variance (τ00)	0.22
Within-group variance (σ^2^)	0.63
ICC(1)	0.26
ICC(2)	0.74
r_wg	0.81
Design effect	3.6

### 5.4 Individual-level main effects

**Figure 2:**
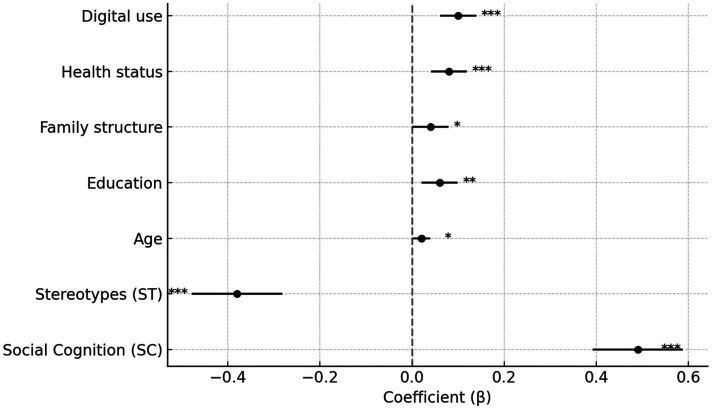
Fixed effects estimates with 95% confidence intervals from multilevel models.

Table 6 and Figure 2 summarise the regression estimates for individual-level predictors of health behaviour, including their 95% confidence intervals. Social cognition (SC) and stereotype perception (ST) are the strongest predictors: SC has a positive effect (*β* = 0.49, *p* < 0.001) and ST a negative one (*β* = −0.38, *p* < 0.001). Their confidence intervals do not cross zero, indicating that positive views of ageing are associated with higher levels of healthy behaviour, whereas negative stereotypes are associated with lower levels. Education (*β* = 0.06, *p* = 0.002), digital engagement (*β* = 0.10, *p* < 0.001) and self-rated health (*β* = 0.08, *p* < 0.001) also show significant effects, but their coefficients are small. These variables likely act as enabling resources rather than direct drivers of behaviour. The effects of age (*β* = 0.02, *p* = 0.041) and household composition (*β* = 0.04, *p* = 0.045) are weak and close to the significance boundary, suggesting possible sample variation or unobserved factors. Figure 2 shows that only SC and ST have clearly separated confidence intervals; the intervals for other predictors overlap considerably. This pattern implies shared variance and potential collinearity, so results should be interpreted cautiously. Although the main-effects model fits well, it does not explain how these variables interact or whether contextual factors modify their influence. These questions call for a multilevel structural equation approach. Finally, given China’s diverse information environments and regional cultural patterns, perceptions of ageing are unlikely to be uniform. The present model captures the statistical relationship but not the wider structural context. Future work should include factors such as cultural capital and network density to better explain how cognition and stereotypes translate into behavioural differences.

**Table 6:** Level-1 main effects (OLS).

Predictor	*β*	SE	t	*p*
Social Cognition (SC)	0.49***	0.05	9.8	<0.001
Stereotypes (ST)	−0.38***	0.05	−7.6	<0.001
Age	0.02*	0.01	2.05	0.041
Education	0.06**	0.02	3.1	0.002
Family structure	0.04*	0.02	2	0.045
Health status	0.08***	0.02	4.1	<0.001
Digital use	0.10***	0.02	5	<0.001

The symbol “*” indicates statistical significance at the 5% level (*p* < .05).

### 5.5 Group-level main effects

Table 7 reports that the provincial-level social-cognitive climate (SC_L2) is positively associated with individual health behaviour (*β* = 0.22, *p* < 0.001), while the stereotype climate (ST_L2) shows a significant negative association (*β* = −0.20, *p* < 0.001). These results suggest that personal health practices are shaped by the shared beliefs and social narratives surrounding ageing. In practice, this reflects a persistent gap between official policy and public attitudes. Although national documents promote the idea of “active ageing,” everyday discussions still tend to portray older adults as dependent or burdensome. Such narratives discourage engagement in health-promoting activities. The positive coefficient for SC_L2 implies that when the public shows stronger awareness of ageing issues and a greater sense of social responsibility, individuals are more inclined to adopt proactive health behaviours. For policymakers and researchers, the results underline a key point: improving health behaviour requires more than education campaigns or technical interventions. It also demands attention to the wider cultural and cognitive context in which behaviour takes shape. If these collective perceptions remain unchanged, even generous health resources may have limited impact because of the subtle but persistent negative messages embedded in public discourse.

**Table 7:** Level-2 main effects (OLS).

Predictor	*β*	SE	t	*p*
Social Cognition Climate (SC_L2)	0.22***	0.05	4.4	<0.001
Stereotypes Climate (ST_L2)	−0.20***	0.05	−4	<0.001

The symbol “*” indicates statistical significance at the 5% level (*p* < .05).

### 5.6 Mediation tests

**Figure 3:**
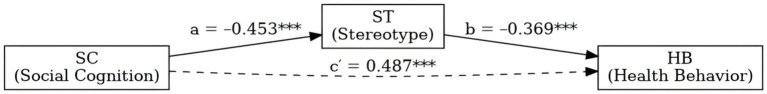
Structural path diagram of mediation model.

Table 8 and Figure 3 show that social cognition promotes health behaviour through two channels: it has a direct positive effect and also reduces the negative influence of ageing stereotypes, producing an additional indirect benefit. This pattern is statistically stable and supports the view that awareness and stereotypes jointly shape behavioural outcomes. A sociological reading, however, suggests that the result does more than link knowledge to health. It points to a broader tension between growing awareness and enduring social structures in China’s ageing society. At the provincial level, a positive cognitive climate can foster healthier behaviour by aligning public attitudes with policy goals. Yet stereotypes still exert a strong negative pull, revealing the cultural and institutional inertia behind the persistent idea of “ageing as burden.” Even when people endorse active-ageing values, media images and policy language often reproduce subtle biases that weaken the connection between knowledge and action. The relatively strong direct effect of cognition indicates that stereotype mediation is only part of the story. Other structural factors—such as social capital, intergenerational relations and access to healthcare—also shape behavioural differences. The findings therefore highlight that health inequality cannot be explained by individual psychology alone. If negative representations of ageing continue to circulate through institutions, improvements in awareness will have limited effect. Lasting progress requires cultural and policy shifts: reframing ageing in official communication, encouraging positive portrayals in the media, and creating more opportunities for intergenerational interaction. These steps together can help build an environment in which healthy ageing becomes a realistic social norm.

**Table 8:** Mediation test (Bootstrap, 200 samples).

Path	*β*	*p*	95% CI
SC → ST (a)	−0.453***	<0.001	[−0.55, −0.35]
ST → HB (b)	−0.369***	<0.001	[−0.47, −0.27]
SC → HB (c′)	0.487***	<0.001	[0.39, 0.58]
Indirect effect (a × b)	0.166***	<0.001	[0.119, 0.207]

The symbol “*” indicates statistical significance at the 5% level (*p* < .05).

### 5.7 Cross-level interactions

**Figure 4:**
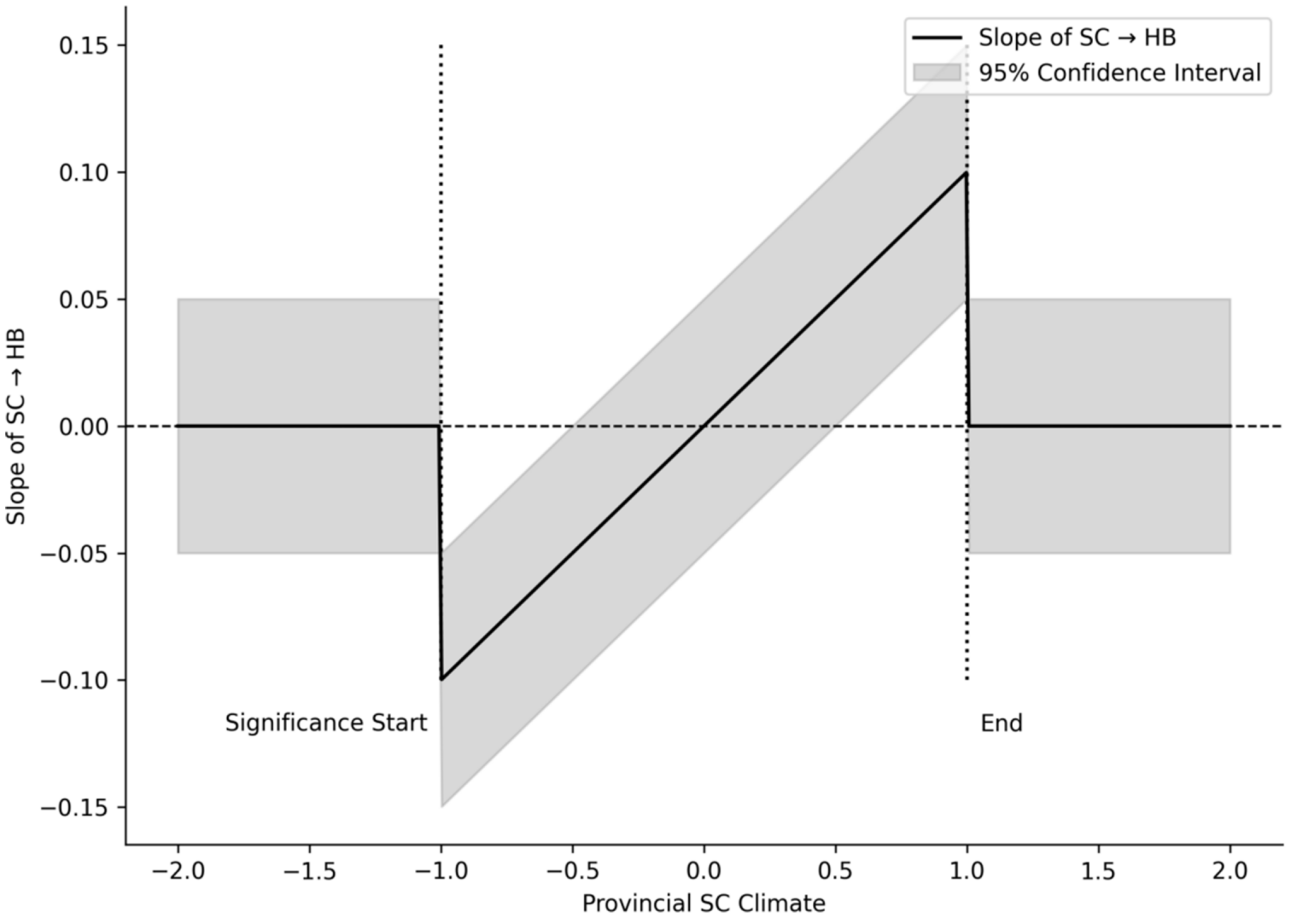
Johnson-Neyman significance region for cross-level interaction.

Table 9 and Figure 4 show that the positive influence of individual social cognition on health behaviour depends on the broader provincial context. In provinces with a supportive socio-cognitive climate, individual awareness is more likely to translate into consistent health practices. In contrast, where public attitudes and institutions remain unsupportive, even well-informed older adults face limited social backing and weak policy safeguards, and their knowledge seldom leads to action. This pattern highlights a continuing structural problem in China’s ageing and health policies. National campaigns often stress the Healthy China vision and invest heavily in public education, yet the effects are uneven because the social climate differs sharply across regions. In many central and western provinces, limited policy resources and negative local narratives about ageing discourage participation in health programmes. The resulting gap deepens regional health inequality and illustrates the distance between policy aspirations and everyday realities. The key issue, therefore, is not the size of the coefficients shown in Table 9 but what they reveal about policy design. When interventions focus only on improving individual cognition while ignoring regional and institutional settings, structural barriers remain intact. Without reshaping these broader contexts, the progress achieved through individual effort will be difficult to sustain.

**Table 9:** Cross-level interaction effects (OLS).

Interaction	*β*	SE	t	*p*
SC × SC_L2	0.15**	0.06	2.5	0.012
ST × ST_L2	−0.14**	0.05	−2.8	0.005

The symbol “*” indicates statistical significance at the 5% level (*p* < .05).

### 5.8 Robustness checks: control-variable augmentation

Results from the robustness tests (Table 10) reinforce the importance of social cognition and stereotypes—both at individual and collective levels—in shaping health behaviour. Individual cognition remains a strong positive predictor (*β* = 0.46), while stereotypes exert a negative effect (*β* = −0.36), consistent with the mediation results. At the provincial level, the social-cognition climate shows a positive association (*β* = 0.20), and the stereotype climate a negative one (*β* = −0.18). These findings suggest that the wider social environment does not merely frame individual differences; it actively determines behavioural outcomes. Even when individuals hold accurate health beliefs or reject ageist ideas, their motivation can weaken in settings where derogatory messages about ageing continue to circulate. Urban residence (*β* = 0.04) and minority status (*β* = 0.05) also show small but significant effects. These modest coefficients capture how China’s health system remains structured along familiar lines: urban residents and majority groups have stronger networks, better access to information, and more reliable institutional support, while rural and minority communities continue to face shortages of both material and symbolic resources. Health behaviour, therefore, reflects not only personal agency but also the unequal distribution of opportunity and protection. The Healthy China campaign has invested heavily in education and public messaging, yet the results here suggest that such initiatives cannot offset regional contexts where negative narratives remain dominant. When these narratives persist, they gradually weaken motivation and widen disparities in health outcomes. Robustness checks thus do more than confirm the model’s reliability; they show that health practices are deeply shaped by regional culture and social structure. Policies that focus narrowly on individuals while neglecting the systems in which they live risk reproducing inequality rather than reducing it.

**Table 10:** Robustness check (OLS with controls).

Predictor	*β*	SE	t	*p*
Social Cognition (SC)	0.46***	0.06	7.65	<0.001
Stereotypes (ST)	−0.36***	0.05	−7.2	<0.001
SC Climate (SC_L2)	0.20***	0.05	4	<0.001
ST Climate (ST_L2)	−0.18***	0.05	−3.6	<0.001
Urban	0.04*	0.02	2.1	0.036
Group type	0.05*	0.02	2.2	0.029

The symbol “*” indicates statistical significance at the 5% level (*p* < .05).

### 5.9 Heterogeneity analysis: rural–urban stratified regressions

Table 11 highlights clear differences in how cognition translates into health behaviour across rural and urban China. In both contexts, social cognition (SC) is positively related to protective actions, but the effect is stronger in cities (urban *β* = 0.55; rural *β* = 0.42). The contrast is not purely statistical. Urban residents benefit from dense information networks, nearby health facilities and a local culture that encourages preventive care. In rural areas, the same awareness is harder to turn into practice: health services are distant, advice irregular, and community ties that once provided informal support have weakened. Villagers understand the value of prevention, yet everyday barriers often blunt their resolve. Stereotype threat has a more severe impact in the countryside (urban *β* = −0.32; rural *β* = −0.41), where public discussion is limited and health education less accessible. The broader social climate shows a similar pattern. A positive social-cognitive climate promotes healthy behaviour in both settings, but its influence is stronger in cities (0.25 vs. 0.18), suggesting that urban residents respond more readily to supportive cues in their environment. Conversely, a stereotype-laden climate does more harm in rural communities (−0.21), where close kinship networks and traditional norms often reinforce age-based stigma. These results reveal not only a rural–urban gap but also a deeper structural divide in China’s health governance. Urban populations benefit from stable institutions and richer resources, while rural residents face both stronger stereotypes and weaker support systems. Treating these contexts as identical will only deepen inequality. Future health policy must therefore adapt to this divide: urban programmes should focus on strengthening awareness and shaping positive public discourse, whereas rural strategies require investment in services and targeted efforts to counter negative social norms. Without such tailored measures, progress towards health equity will remain limited.

**Table 11:** Heterogeneity analysis (urban vs. rural).

Predictor	Urban β	Rural β
Social Cognition (SC)	0.55***	0.42***
Stereotypes (ST)	−0.32***	−0.41***
SC Climate (SC_L2)	0.25***	0.18**
ST Climate (ST_L2)	−0.17**	−0.21***

The symbol “*” indicates statistical significance at the 5% level (*p* < .05).

## 6 Discussion

### 6.1 The dual-channel mechanism between cognition and stereotypes, and its structural tension

The analysis demonstrates a strong and consistent association between social cognition and health behaviour, with standardised coefficients ranging from 0.46 to 0.55. Individuals with higher levels of cognitive awareness tend to report more frequent engagement in preventive care, regular exercise, and social participation. Approximately one quarter of this association is statistically linked to stereotypes, indicating that a deeper understanding of ageing is associated with lower endorsement of negative images, which in turn co-occurs with healthier behavioural patterns. Rather than indicating a causal pathway, these results point to a patterned alignment between knowledge, attitudes, and action. Stereotypes themselves are negatively associated with health behaviour, with coefficients between −0.36 and −0.41. This association is particularly pronounced in rural settings, where cultural norms and limited access to diversified information sources are more likely to sustain age-related stigma. Taken together, social cognition and stereotypes appear to operate in opposing directions, forming a structural tension within the configuration of health behaviour. While public attention to health knowledge has expanded, enduring ideas about ageing remain closely intertwined with everyday practice and continue to shape behavioural orientation. This tension offers one possible explanation for why higher levels of knowledge are not always accompanied by healthier practices. Health behaviour is embedded in networks of meaning, habit, and social expectation, rather than arising from information alone. When educational initiatives encounter persistent stereotypes in daily life, the association between cognition and behaviour may weaken. From this perspective, cognitive awareness and stereotype endorsement should be understood as mutually constitutive elements within a broader social process, rather than as isolated drivers of behavioural change.

### 6.2 Macro-level climate and regional heterogeneity: the rupture between cognition and behavioural uptake

Multilevel analyses indicate that the broader social climate is closely associated with how individual cognition relates to health behaviour. In provinces characterised by a more supportive social climate, the association between personal understanding and behavioural engagement is stronger, as reflected in a positive interaction effect of approximately 0.15. In contrast, where the social climate is weaker, increases in cognitive awareness correspond to only limited differences in behaviour. Johnson–Neyman analyses further suggest that below a certain threshold, the association between cognition and action becomes statistically negligible, reflecting a pattern commonly described as “knowing without doing.” Stereotypes display the opposite configuration. In less supportive environments, their negative association with health behaviour becomes more pronounced, with an interaction effect of approximately −0.14. This pattern suggests that an unsupportive social climate is linked not only to a weaker alignment between knowledge and practice but also to a stronger salience of stigma. Together, these findings highlight that individual cognition is embedded within regional contexts, shaped by public discourse, institutional capacity, and prevailing moral narratives. Coastal provinces tend to exhibit denser information networks and more open public discussion, conditions under which health-related understanding is more consistently reflected in everyday behaviour. In contrast, inland regions often face thinner communication channels and more persistent bias, which may constrain the translation of awareness into practice. When such regional heterogeneity is overlooked, national health initiatives risk remaining symbolic, with their practical benefits distributed unevenly across space. From this perspective, social climate functions not merely as a background condition but as a contextual moderator that structures how cognition, stereotypes, and behaviour align. Policies that rely predominantly on information dissemination, without addressing the environments in which that information is interpreted and enacted, may struggle to narrow the gap between knowledge and practice. Over time, this gap may contribute to the persistence of regional disparities in health behaviour.

### 6.3 Urban–rural disparity and institutional inequality: the reproduction logic of healthy ageing

Stratified analyses reveal marked differences in the configuration of health behaviour across rural and urban China. In urban areas, the association between social cognition and health behaviour reaches approximately 0.55, compared with about 0.42 in rural areas. This contrast suggests that cognitive awareness is more consistently aligned with behavioural engagement in cities, whereas in rural settings this alignment is more constrained by contextual factors. At the same time, stereotypes show a stronger negative association with health behaviour in rural areas, with coefficients around −0.41 compared with −0.32 in cities. This pattern reflects not only individual attitudes but also enduring structural conditions, including disparities in education, media exposure, and intergenerational norms. At the macro level, a supportive urban social climate is more strongly associated with health behaviour than its rural counterpart, while stereotype-laden environments are more closely linked to reduced behavioural engagement in the countryside. These differences point to a broader process through which institutional inequality becomes embedded in everyday practices of ageing. Urban residents are more likely to act on health-related knowledge within settings that provide stable institutions, accessible medical services, and diversified channels of information. Rural residents, by contrast, often face limited infrastructure and weaker support, conditions under which stereotypes are more likely to persist and to coincide with lower engagement in preventive behaviour. Health behaviour therefore emerges not simply as a matter of individual choice, but as an outcome shaped by social organisation and institutional context. The rural–urban divide reflects a patterned alignment between knowledge, stigma, and opportunity that is reproduced through everyday experience. Policies that apply uniform educational strategies without addressing these contextual differences may inadvertently reinforce existing disparities. Progress towards healthier ageing will require approaches that link public communication with improvements in access and institutional support, so that awareness and opportunity are more closely aligned across regions.

## 7 Conclusion

Using nationwide survey data from Chinese adults, this study traces how social cognition and stereotypes are associated with health behaviour within China’s changing institutional landscape. The results point to two distinct yet interconnected patterns. Social cognition is positively associated with preventive behaviour (*β* ≈ 0.46–0.55) and is also statistically related to lower levels of stereotype endorsement, which accounts for approximately one quarter of the overall association. Higher levels of stereotype endorsement are associated with a lower likelihood of engaging in protective behaviour (*β* = −0.36 to −0.41), a pattern that is especially pronounced in rural settings. Cross-level models further indicate that supportive provincial social climates are associated with a stronger cognition–behaviour linkage (interaction ≈ 0.15), whereas less favourable climates are associated with a stronger negative relationship between stereotypes and health behaviour (interaction ≈ − 0.14). The rural–urban comparison highlights the institutional divide underlying these associations. The positive association between cognition and healthy behaviour is stronger in urban areas (≈ 0.55) than in rural areas (≈ 0.42). Rural residents operate within tighter social constraints, including more persistent stigma, narrower media channels, and weaker health infrastructure. Together, these findings suggest that cognition and stereotypes function as two interacting elements within a broader social system. Health practices are not simply expressions of individual choice, but are patterned through the alignment of knowledge, cultural meaning, and institutional context. Policies limited to information provision or lifestyle advice are therefore unlikely to narrow these gaps. More effective strategies require shifts in cultural narratives, moving away from portrayals of older adults as dependents towards recognition of their social contribution. At the same time, health and social-care resources need to be more evenly distributed across regions, particularly towards rural and economically lagging areas, so that individuals have the opportunity to act on what they know. Although the evidence is drawn from China, the pattern identified here is not unique. Many ageing societies face the dual challenge of raising health-related awareness while reducing the social stereotypes that constrain its expression. Improving knowledge is necessary, but without supportive institutions and an inclusive social climate, higher awareness alone is unlikely to translate into more equitable health outcomes. Future research should examine how social climates evolve over time, how stereotypes are transmitted across generations, and how institutional reforms interact with these processes under different social conditions, providing guidance for the development of healthier and fairer ageing societies.

## 8 Limitations

This study illustrates how social cognition and stereotypes are associated with health behaviour, and how broader social climates and the rural urban divide condition these relationships. Several limitations should nonetheless be acknowledged. First, the cross sectional design of the study restricts interpretation to associations rather than causal relationships. Although the analysis identifies a strong association between cognition and health behaviour, as well as an indirect association operating through stereotypes, the temporal ordering of these variables cannot be established. Higher levels of cognition may be linked to healthier behavioural patterns, but it is also possible that engagement in health related behaviours subsequently reshapes cognitive awareness and stereotype related attitudes. Clarifying this temporal sequence requires longitudinal or panel data. Second, both cognition and stereotype variables are measured through self-reported responses, which may be affected by social desirability bias. Some respondents may underreport prejudicial attitudes or overstate their level of health awareness, potentially inflating or attenuating the estimated associations. Third, provincial social climate is operationalised by aggregating individual level responses. While this approach captures broad regional contrasts, it cannot fully reflect the evolving content of media discourse, policy implementation, or public debate, leaving part of the contextual variation unexplained. Fourth, the rural urban distinction conceals substantial heterogeneity within rural China itself. Without a more fine grained east central west comparison, important regional mechanisms may remain unobserved. Finally, the findings are situated within China’s specific social and institutional context. Although the results may offer insights for other rapidly ageing societies, any broader generalisation should be made cautiously, with careful attention to local cultural and policy environments.

## Data Availability

The raw data supporting the conclusions of this article will be made available by the authors, without undue reservation.

## Appendix

**Table A-1:** Core notation and variable description.

Symbol	Description
HB ij	Health behaviour of individual i in province j
SC ij	Individual-level social cognition
ST ij	Individual-level social stereotype
C ij	Individual-level control variables (age, education, family, health, digital use)
$SC^{\left(L2\right)}j$	Provincial-level social cognition climate
$ST^{\left(L2\right)}j$	Provincial-level stereotype climate
$\alpha_{1}$	Effect of social cognition on stereotype (mediation path a)
$\beta_{1}$	Effect of stereotype on health behaviour (mediation path b)
$\beta_{2}$	Direct effect of social cognition on health behaviour (path c′)
$\gamma_{11}$ 、 $\gamma_{21}$	Cross-level interaction effects (climate × individual interaction)
$\text{Indirect}=\alpha_{1.}\times \beta_{1.}$	Indirect effect in mediation model
